# Molecular docking‐assisted screening reveals tannic acid as a natural protein disulphide isomerase inhibitor with antiplatelet and antithrombotic activities

**DOI:** 10.1111/jcmm.16043

**Published:** 2020-10-30

**Authors:** Lijie Ren, Tao You, Qing Li, Guona Chen, Ziting Liu, Xuefei Zhao, Yinyan Wang, Lei Wang, Yi Wu, Chaojun Tang, Li Zhu

**Affiliations:** ^1^ Cyrus Tang Hematology Center Collaborative Innovation Center of Hematology Suzhou Key Laboratory of Thrombosis and Vascular Diseases State Key Laboratory of Radiation Medicine and Protection Soochow University Suzhou China; ^2^ National Clinical Research Center for Hematologic Diseases the First Affiliated Hospital of Soochow University Suzhou China; ^3^ Jiangsu Institute of Hematology Key Laboratory of Thrombosis and Hemostasis of Ministry of Health The First Affiliated Hospital of Soochow University Suzhou China; ^4^ Department of Cardiology The Second Affiliated Hospital of Soochow University Suzhou China

**Keywords:** platelets, protein disulphide isomerase, tannic acid, thrombosis

## Abstract

Protein disulphide isomerase (PDI) promotes platelet activation and constitutes a novel antithrombotic target. In this study, we reported that a PDI‐binding plant polyphenol, tannic acid (TA), inhibits PDI activity, platelet activation and thrombus formation. Molecular docking using plant polyphenols from dietary sources with cardiovascular benefits revealed TA as the most potent binding molecule with PDI active centre. Surface plasmon resonance demonstrated that TA bound PDI with high affinity. Using Di‐eosin‐glutathione disulphide fluorescence assay and PDI assay kit, we showed that TA inhibited PDI activity. In isolated platelets, TA inhibited platelet aggregation stimulated by either GPVI or ITAM pathway agonists. Flow cytometry showed that TA inhibited thrombin‐ or CRP‐stimulated platelet activation, as reflected by reduced granule secretion and integrin activation. TA also reduced platelet spreading on immobilized fibrinogen and platelet adhesion under flow conditions. In a laser‐induced vascular injury mouse model, intraperitoneal injection of TA significantly decreased the size of cremaster arteriole thrombi. No prolongation of mouse jugular vein and tail‐bleeding time was observed after TA administration. Therefore, we identified TA from natural polyphenols as a novel inhibitor of PDI function. TA inhibits platelet activation and thrombus formation, suggesting it as a potential antithrombotic agent.

## 1 INTRODUCTION

Protein disulphide isomerase (PDI) is the first identified member of protein disulphide isomerases, a protein family that catalyses the turnover of disulphide bonds and regulates protein folding.^1^ Accumulating studies showed that PDI regulates platelet activation and thrombus formation via extracellular reductase activities. The structure of PDI contains four thioredoxin domains, namely a, b, b′ and a′. The CGHC redox‐active motifs in a and a′ domains of PDI is the key structure for its catalytic function, and the b′ domain with a hydrophobic pocket mediates binding with substrates including integrins, coagulation factors and adhesive proteins. PDI is released extracellularly during platelet activation and binds electrostatically to integrin α_IIb_β_3_, catalysing the reduction and exchange of disulphide bonds on integrin surface. Structural change of integrin α_IIb_β_3_ to high‐affinity states then promotes intracellular platelet signalling and thrombus formation. Besides, PDI can initiate coagulation by activating factor V^2^ and tissue factor.^3^ By activating neutrophil integrin α_M_β_2_ and endothelial integrin α_V_β_3_, PDI is involved in vascular inflammation and homeostasis. The emerging role of PDI in platelet activation renders it a versatile target in thrombotic diseases.^4^

Growing efforts have been made to seek potential antithrombotic agents that inhibit PDI activity, spectacularly in the forms of monoclonal antibodies^3^, ^5^, ^6^ and small molecular compounds.^7^, ^8^ Quercetin‐3‐rutinoside (rutin) was identified from molecule screening as a natural inhibitor of PDI that inhibits platelet activation. In vivo studies indicated that rutin exhibited antithrombotic effects by targeting extracellular PDI without affecting hemostasis in mice.^9^ A pilot clinical trial showed that oral administration of isoquercetin, the 3‐O‐glucoside of quercetin, inhibited PDI‐mediated platelet factor Va activation and platelet‐dependent thrombin generation.^2^ A later phase II multi‐centre clinical trial (NCT02195232) showed that isoquercetin administration reduced hypercoagulability in patients with advanced cancer without increasing bleeding risk.^10^

Large‐scale prospective studies revealed the beneficial role of a healthy diet in preventing and controlling cardiovascular diseases (CVD).^11^ Intake of the Mediterranean diet rich in plant polyphenols has been associated with reduced cardiovascular mortality.^12^, ^13^, ^14^, ^15^ Besides their well‐known anti‐oxidative activities, emerging evidence suggests that plant polyphenols may inhibit enzymes via regulating different pathways underlying CVD, including extracellular matrix degradation, inflammatory response and cell death. Besides, plant polyphenols have been reported as a natural source of antiplatelet and antithrombotic agents.^16^, ^17^, ^18^ Previous studies using in vitro screening have demonstrated that several plant polyphenols, including rutin and isoquercetin, inhibits PDI activity and thrombus formation. Considering their distribution and abundance in daily diets, polyphenols may constitute a class of safe candidates with PDI inhibitory activity.

In this study, we aimed to screen polyphenols from food and beverages with potential cardiovascular benefits for a candidate that interacts with PDI. Tannic acid (TA, gallotannin) was identified as the most potent binding partner with PDI. TA inhibits PDI activity, platelet activation and thrombus formation in vivo. These results showed the role of TA as a novel natural inhibitor of PDI with antithrombotic potency, highlighting its potential application in managing thrombotic diseases.

## 2 MATERIALS AND METHODS

### 2.1 Animals

Male C57BL/6 mice were purchased from the laboratory animal core facility of Soochow University. All animal protocols were in accordance with the Guide for the Care and Use of Laboratory Animals of the US National Institutes of Health and were approved by the University Committee on Animal Care of Soochow University.

### 2.2 Reagents and antibodies

Tannic acid was purchased from Sigma‐Aldrich. Collagen‐related peptide (CRP) was a gift from Dr Yukio Ozaki at Yamanashi University. Human PDI recombinant protein, ERp72 and ERp57 were gifts from Dr Yi Wu at Soochow University. 3‐(*N*‐Maleimidylpropionyl) biocytin was purchased from Molecular Probes. CD62P and PAC‐1 antibodies were purchased from BD Biosciences. PROTEOSTAT PDI Assay Kit was purchased from Enzo Life Sciences.

### 2.3 Blood collection and platelet preparation

Fasting venous blood was obtained from healthy donors and immediately anticoagulated with ACD buffer. Washed and gel‐filtered platelets were prepared as described previously.^19^, ^20^ Procedures utilizing human samples were approved by the University Ethical Committee of Soochow University and were in accordance with the Declaration of Helsinki. Informed consent was obtained from all participants.

### 2.4 Platelet aggregation

Platelet aggregation was performed using a Chrono‐Log aggregometer. Gel‐filtered human platelets were incubated with vehicle (saline) or TA in a glass cuvette for 10 minutes at 37°C. CaCl_2_ (1 mmol/L) and agonists were added to the platelet suspension with stirring at 900 rpm. Aggregation was measured by a turbidity method.

### 2.5 Flow cytometry

Gel‐filtered human platelets (5 × 10^7^/mL) were treated with TA or vehicle and incubated with FITC‐conjugated PAC‐1 and PE‐conjugated CD62P antibody, respectively. Platelets were stimulated using CRP (1 μg/mL) or thrombin (0.2 U/mL) for 15 minutes and resuspended in PBS. Besides, a TA toxicity assay was performed following the procedure described in the manual of the Annexin V FITC APOPTOSIS Detection KIT. Fluorescence intensity was measured using Flow Cytometer.

### 2.6 Platelet spreading on immobilized fibrinogen

Gel‐filtered human platelets (5 × 10^7^/mL) were treated with TA (10, 30 and 50 μmol/L) or vehicle for 10 minutes, seeded on the fibrinogen (10 μg/mL)‐coated coverslips and allowed to spread for 60 minutes. Adherent platelets were labelled using TRITC‐labelled phalloidin and observed using a confocal microscope. Platelet surface areas were assessed using ImageJ software.

### 2.7 Clot retraction

Gel‐filtered platelets were pre‐incubated with TA (30 μmol/L) or vehicle for 10 minutes at 37°C. Then, the platelets were transferred to the siliconized glass tubes and stimulated with fibrinogen (2 mg/mL) and thrombin (1 U/mL). The clot retraction was recorded at indicated time point. The areas of platelet clots were calculated using ImageJ software.

### 2.8 PDI activity assay

Recombinant human PDI (20 nmol/L) was incubated with TA in an assay buffer (0.1 mol/L PBS, 2 mmol/L EDTA, pH 7.0) for 5 minutes at room temperature. In the presence of DTT (5 μmol/L), Di‐E‐GSSG (150 nmol/L) was incubated with the samples and the increase in fluorescence (emission/excitation 545/525 nm) was recorded using a spectrophotometer as described previously.^21^, ^22^ The degree of inhibition was relative to the total amount of EGSH formed over the time of the assay. Besides, a PDI‐catalysed reduction of insulin assay was performed following the procedure described in the manual of the PDI Assay Kit.

### 2.9 3‐(*N*‐maleimidylpropionyl) biocytin (MPB) labelling of platelets

MPB labelling of platelets was performed as previously described.^22^ After treatment with TA (30 μmol/L), platelets were stimulated by 0.05 U/L thrombin and labelled with MPB (100 μmol/L, for 30 minutes) at room temperature. Samples were washed with EDTA (2 mmol/L) and lysed using RIPA buffer. Samples were separated using 10% SDS‐PAGE and incubated in IRDye 800 fluorescent‐conjugated streptavidin for 1 hour. Densitometry analysis was performed using the ImageJ software.

### 2.10 Molecular docking

A customized polyphenol library was derived from natural dietary sources including green tea, red wine, olive oil and coffee. Structures of all polyphenol were obtained from the PubChem database (https://pubchem.ncbi.nlm.nih.gov/).^23^ Structures of oxidized (4EL1) or reduced (4EKZ) human PDI were downloaded from the RCSB PDB server (https://www.rcsb.org/structure/4EKZ).^24^, ^25^ SystemDock server (http://systemsdock.unit.oist.jp/iddp/home/index) ^26^ was used to evaluate the interaction between polyphenols and PDI molecules. The structures of polyphenol compounds and PDI were selected as ligands and receptors for systemDock input. For docking with the active centres and substrate‐binding site in the a, a′ and b′ domain in 4EL1, the spatial coordinates of docking centres were set at 31.948_5.963_47.631, 22.8_18.243_8.778 and 4.396_2.236_18.784, respectively. For the docking with 4EKZ, the coordinates were −18.811_−18.795_4.187, −10.6_−43.961_−1.927 and −14.232_−49.944_15.588. The output of docking scores (p*K*_d_/p*K*_i_) was used to quantify the molecular interaction. A heat map visualization of binding scores was performed using the HEML software (The CUCKOO Working Group, Huazhong University of Science and Technology, Wuhan, Hubei, China).^27^

### 2.11 Adhesion of platelets under flow conditions

Platelet adhesion under flow conditions was measured using a BioFlux200™ flow chamber system (Fluxion Biosciences Inc). Channels were coated with type I collagen (100 µg/mL) for 1 hour at RT. Sodium citrate‐anticoagulated human whole blood was pre‐incubated with TA (50 μmol/L) or saline, labelled with calcein‐AM (10 μmol/L) for 30 minutes, and perfused through the channels at 10 dyne/cm^2^. Adherent platelets were recorded and quantified.

### 2.12 Laser injury‐induced thrombosis

Tannic acid (5 mg/kg) was intraperitoneally injected (ip) 30 minutes before the vascular injury.^28^ Briefly, mice were anaesthetized by pentobarbital, and 3,3′‐dihexyloxacarbocyanine iodide (DIOC6) was infused through the jugular vein. Cremaster arterioles with diameters (30‐50 μm) were irritated by a pulsatile laser. Videos were captured (Video S1 and S2). The geometry of the thrombus was recorded according to the fluorescence intensity above the background.

### 2.13 Tail and jugular vein bleeding time

Tail‐bleeding time was evaluated as previously described.^29^ Briefly, after administration with TA (5 mg/kg), the distal 5 mm of the tail was cut, and the bleeding time was recorded. For the jugular bleeding assay, the jugular veins were exposed. A 28G needle was advanced to introduce a transmural injury on the posterior wall of the jugular vein. Time to bleeding cessation was recorded.

### 2.14 Surface plasmon resonance

Recombinant PDI was diluted in running buffer (PBS 20 nmol/L HEPES and 0.15 mol/L NaCl, pH 7.4, 25°C) and fixed on the flow cell 1 (FL1) of the L1 sensor chip at a flow rate of 10 μL/min with a coupling amount of 5000 RU. TA (0, 0.0625, 0.125, 0.25, 0.5 and 1 μg/mL) was injected into the flow cell at a flow rate of 30 μL/min for 180 seconds and dissociated 7200 in increasing order of concentration. The binding reaction curve of TA to PDI was obtained by the analysis using BIA evaluation software.

### 2.15 Statistics

Statistical analyses were performed using the Prism 8.0 software (Graphpad). Data were presented as means ± standard deviation (SD). Comparisons among multiple groups were performed using one‐way analysis of variance followed by Dunnett's post hoc test. To compare the difference between two groups, two‐tailed unpaired Student's *t* test was used.

## 3 RESULTS

### 3.1 TA binds PDI molecule with high affinity

**FIGURE 1:**
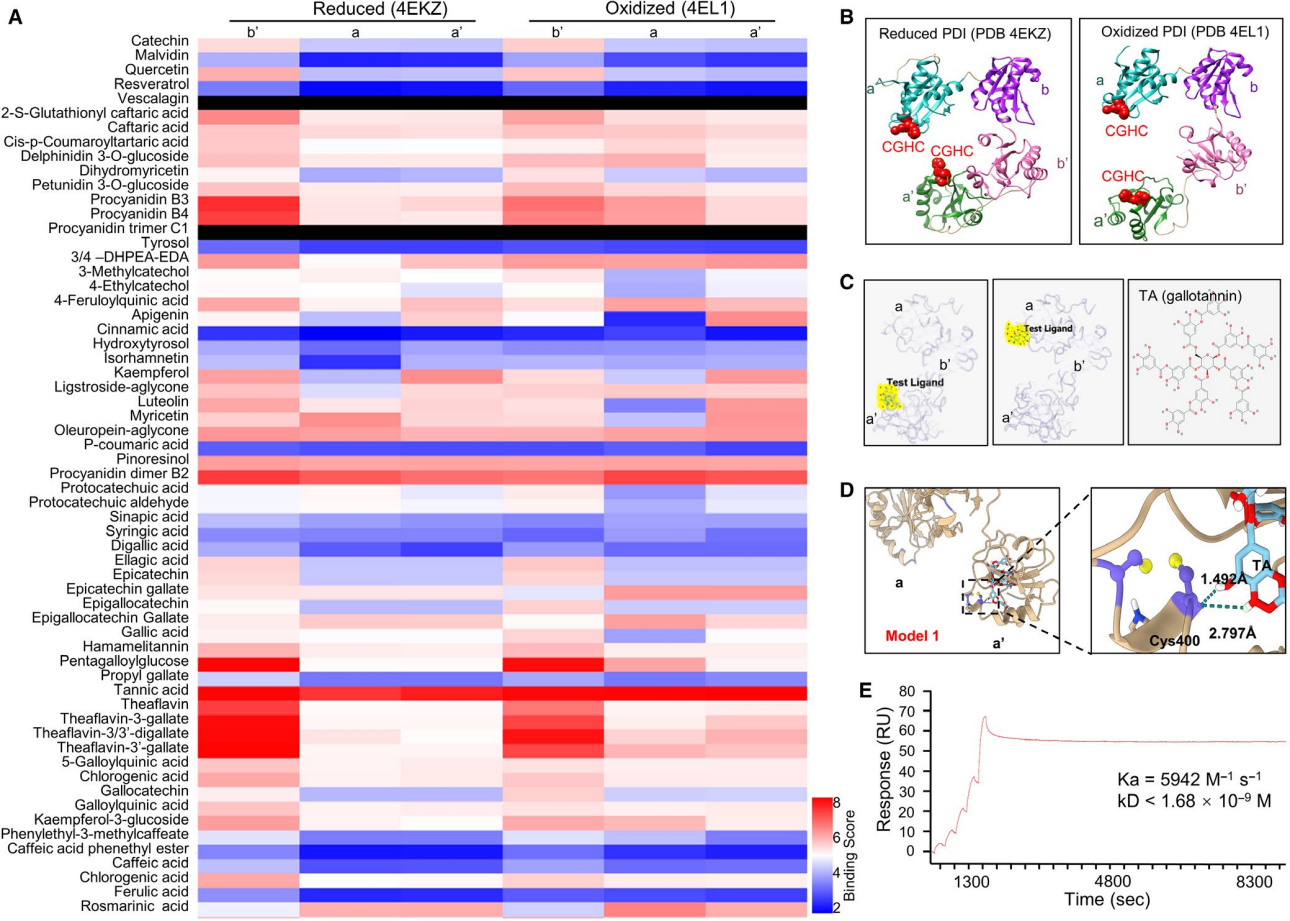
Tannic acid binds PDI molecule with high affinity. SystemsDock website server was used to simulate the interaction between TA and PDI molecular at different oxidative status. A, Heat map of 61 plant‐derived polyphenolic compounds that potentially bind to different domains (b′, a and a′) of reduced (left three columns) or oxidized (right three columns) PDI molecules. Colour bars reflects the rank of binding scores. B, The protein structure of human PDI at reduced and oxidized status was obtained from the PDB database. Red spheres, CGHC active centre. C, Molecular docking results of TA (yellow) binding to the a′ (left panel) and a (middle panel) domain of PDI molecule (grey) from systemsDock and two‐dimensional molecular structure of TA (right panel). Red dot in TA structure, hydroxyl groups. D, Molecular simulation result of TA binding with the a′ domain active centre. Purple structures, the cysteine residues Cys397 and Cys400 at the CGHC active site in the a′ domain of PDI. Yellow sphere, hydroxyl group. The green dotted line in the right panel, hydrogen bond formed between Cys400 and TA. E, Binding of soluble TA in flow with immobilized PDI on biochip was quantified using the surface plasmon resonance assay. SPR sensorgram (red line) showed a strong binding between TA and PDI

A customized polyphenol library from beverages with reported cardiovascular benefits, including coffee, green tea, olive oil and red wines, was evaluated using systemDock web server. The predicted binding affinity of each compound with PDI was depicted in a heat map (Figure 1A). The a, a′ and b′ domain of oxidized or reduce human PDI molecule structures obtained from the PDB database were selected as docking centres (Figure 1B). Conservation between mouse and human PDI was displayed by sequence alignment (Figure S1A). Machine learning (docK‐IN, Random Forest)‐assisted ranking^30^ showed several compounds with high binding potential with either the active centre or substrate‐binding pocket of PDI. Notably, TA achieved the highest binding score with both sites (Figure 1A). To detect the model of interaction between TA and PDI, further docking site simulation was conducted, suggesting that TA may bind the CGHC enzymatic centres of both reduced and oxidized PDI, indicating its potential effect on PDI catalytic activity (Figure 1C,D). Inter‐molecular space estimation suggested two hydrogen bonds forming between TA and the cysteine residue in PDI structure. To validate the physical association of TA with PDI, we used surface plasmon resonance (SPR) to examine the binding of TA and recombinant human PDI in vitro. The real‐time and high sensitive approach^31^ allowed us to detect that TA bound PDI molecules with a binding time of 180 seconds and a dissociation time of 7200 seconds (Figure 1E). The binding constant *K*_a_ was 5942 (mol/L)/s and *k*_D_ was below 1.68 × 10^−9^ mol/L. The binding kinetic parameters suggested that TA binds PDI with a high affinity, while the interaction between TA and PDI was nearly irreversible. These results suggest that TA binds the PDI molecule at its active centre.

### 3.2 TA inhibits PDI reductase activity in vitro and reduces platelet surface thiol generation

**FIGURE 2:**
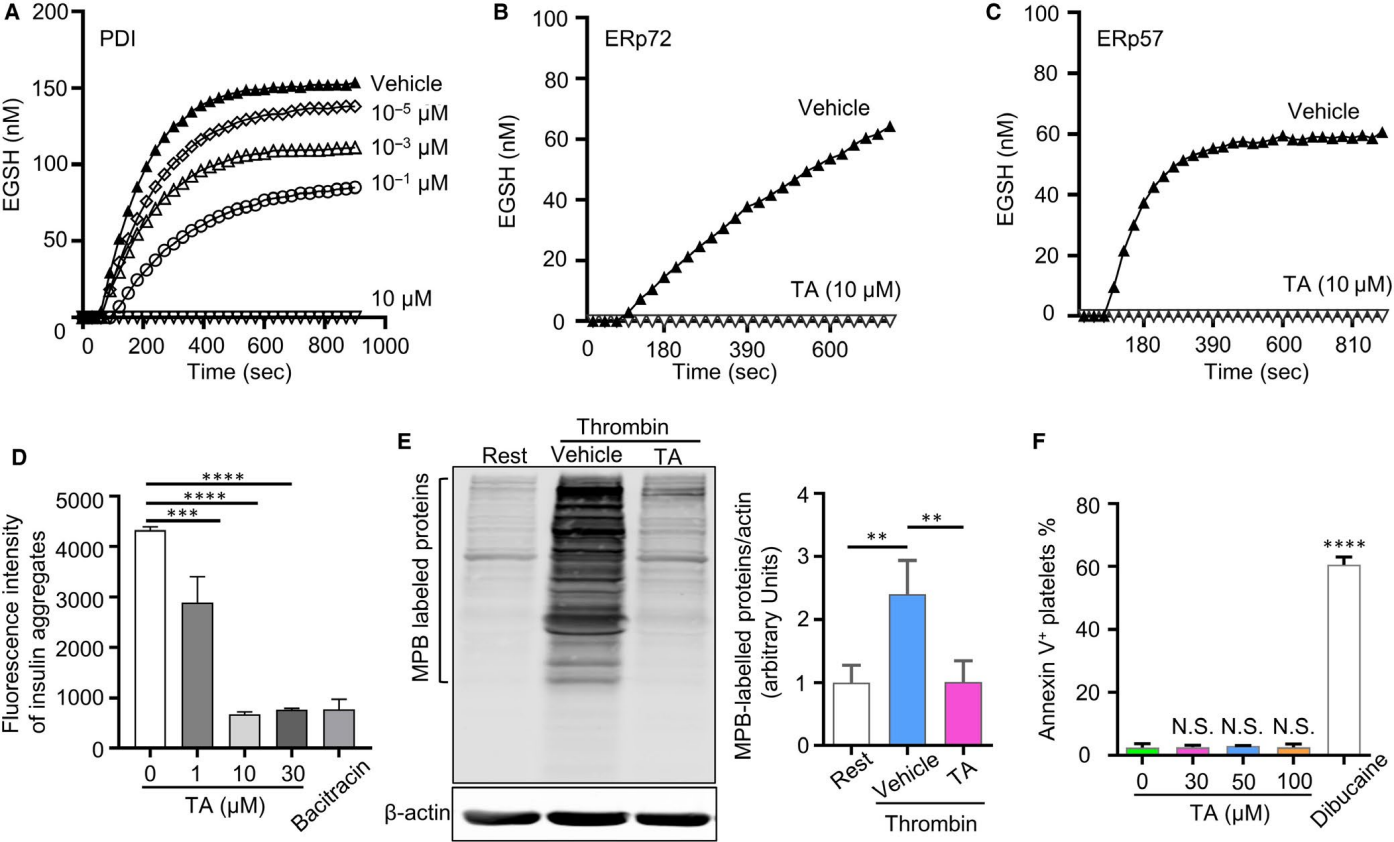
TA inhibits PDI reductase activity in vitro and reduces platelet surface thiol generation. A‐C, Inhibitory effects of TA on PDI activity were measured by the Di‐E‐GSSG assay. Recombinant human PDI, ERP72 or ERP57 (20 nmol/L) were pre‐incubated with TA, saline (vehicle) or Di‐E‐GSSG for 10 min. The reductase activity was measured by monitoring the fluorescence intensity after adding DTT (5 μmol/L). The amount of EGSH (nmol/L) formed from Di‐E‐GSSG in the presence of enzyme over 900 s is shown. D, PDI reduction activity in the presence of TA (1, 10 and 30 μmol/L) was measured by PROTEOSTAT PDI assay kit. Bacitracin (1 mmol/L) was used as a positive PDI inhibition control. Results presented were the mean ± SD of triplicates of one representative experiment. Ordinary one‐way ANOVA, ***P < .001, ****P < .0001. E, MPB‐labelled platelet surface thiol after stimulated by thrombin (0.05 U/mL) in TA (30 μmol/L)‐ or saline‐treated groups (vehicle). The right panel showed platelet surface free sulfhydryl levels. N > 3 per group. Data were presented as means ± SD. Ordinary one‐way ANOVA, **P < .01. F, Annexin V assay was used to determine the toxicity of TA on platelets. N = 3 per group. Data were presented as means ± SD. ****P < .0001, NS, no statistical significance

To determine the effect of TA on PDI activity, the in vitro disulphide reductase assay using di‐eosin glutathione disulphide (di‐E‐GSSG) that comprises two adjacent eosin moieties coupled to an oxidized glutathione disulphide was performed. As shown in Figure 2A, the amount of EGSH (nmol/L) formed from Di‐E‐GSSG in the presence of recombinant PDI was dose‐dependently reduced by TA, indicating the inhibition of PDI reductase activity. Similarly, TA impaired the reductase activity of other platelet PDI family members ERP57 and ERP72 that are involved in thrombus formation (Figure 2B,C).^1^ Moreover, in parallel with the inhibitory effects on PDI reductase activity using di‐E‐GSSG assay, TA also inhibited PDI‐catalysed reduction of insulin (Figure 2D).

As PDI mediates the formation of free thiols in key activation receptors on platelet surface and promotes platelet activation,^32^ we next examined whether TA inhibits the function of PDI on human platelet surface. Platelets were stimulated with thrombin, and membrane thiols were labelled with 3‐(*N*‐maleimidylpropionyl) biocytin (MPB). Immunoblotting of MPB‐labelled proteins showed that treatment with TA (30 μmol/L) reduced the thrombin (0.05 U/mL)‐stimulated generation of platelet membrane thiols almost to the basal level of the resting platelets (Figure 2E).

Antiplatelet agents may induce platelet apoptosis, which compromises their safe use as antithrombotic drugs.^33^ To estimate the potential effect of TA on platelet apoptosis, we evaluated platelet phosphatidylserine (PS) externalization after incubation with TA by flow cytometric analysis of Annexin V as the extent of cellular apoptosis can be measured according to the level of cell surface PS that potently binds Annexin V. Compared with the vehicle, TA (30‐100 μmol/L) did not alter the proportion of Annexin V^+^ platelets. In contrast, the treatment of dibucaine led to a 24‐fold increase in the population of apoptotic platelets (Figure 2F). Therefore, TA, within the tested concentration ranges, does not induce platelet apoptosis.

### 3.3 TA inhibits platelet aggregation, integrin α_IIb_β_3_ activation and P‐selectin expression

**FIGURE 3:**
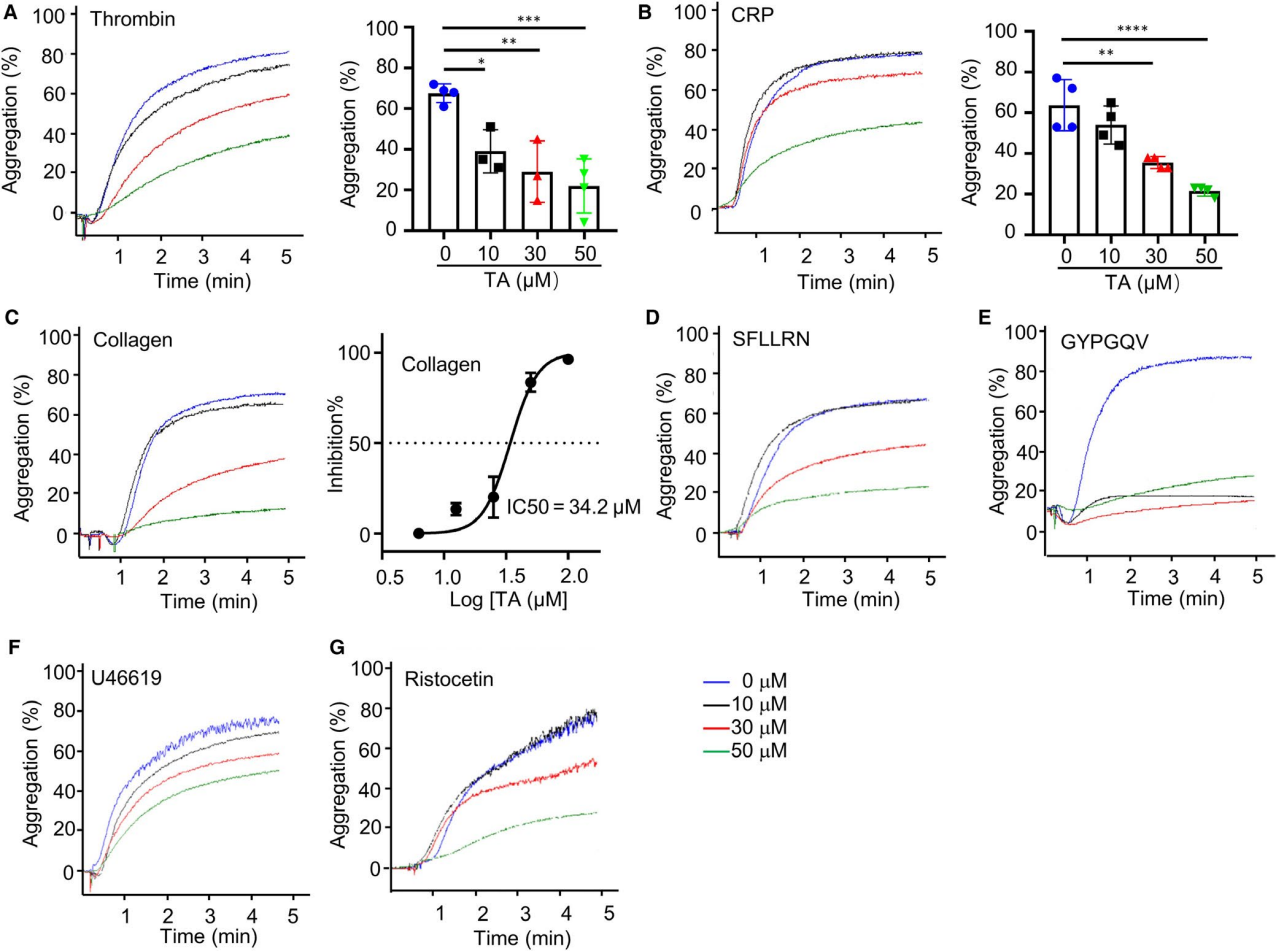
TA inhibits platelet aggregation. Gel‐filtered human platelets were incubated with saline (vehicle) or TA (10, 30 and 50 μmol/L) at 37°C for 10 min. Platelet aggregation stimulated by thrombin (0.05 U/mL) (A), CRP (2 μg/mL) (B) or collagen (2 μg/mL) (C) was recorded and quantified. The IC50 of TA on collagen‐stimulated platelet aggregation was calculated. N = 3. Platelet aggregation stimulated by SFLLRN (50 μmol/L) (D), GYPGQV (1000 μmol/L) (E), U46619 (1 μmol/L) (F) or ristocetin (1 mg/mL) (G) was also measured

Given the essential role of PDI in platelet function and the inhibition of PDI by TA, we next asked whether TA inhibits platelet function using in vitro aggregation assay. Compared with the vehicle, pre‐treatment with 50 μmol/L TA reduced thrombin (0.05 U/mL)‐ and CRP‐stimulated platelet aggregation by 45% and 42%, respectively (Figure 3A,B). Similarly, TA inhibited platelet aggregation stimulated by 2 μg/mL collagen with an IC_50_ of 34.2 μmol/L (Figure 3C). Moreover, TA reduced platelet aggregation stimulated by multiple agonists, including SFLLRN, GYPGQV, U46619 and ristocetin (Figure 3D‐G), suggesting that the inhibitory effect of TA on platelet aggregation was not confined to a single pathway.

**FIGURE 4:**
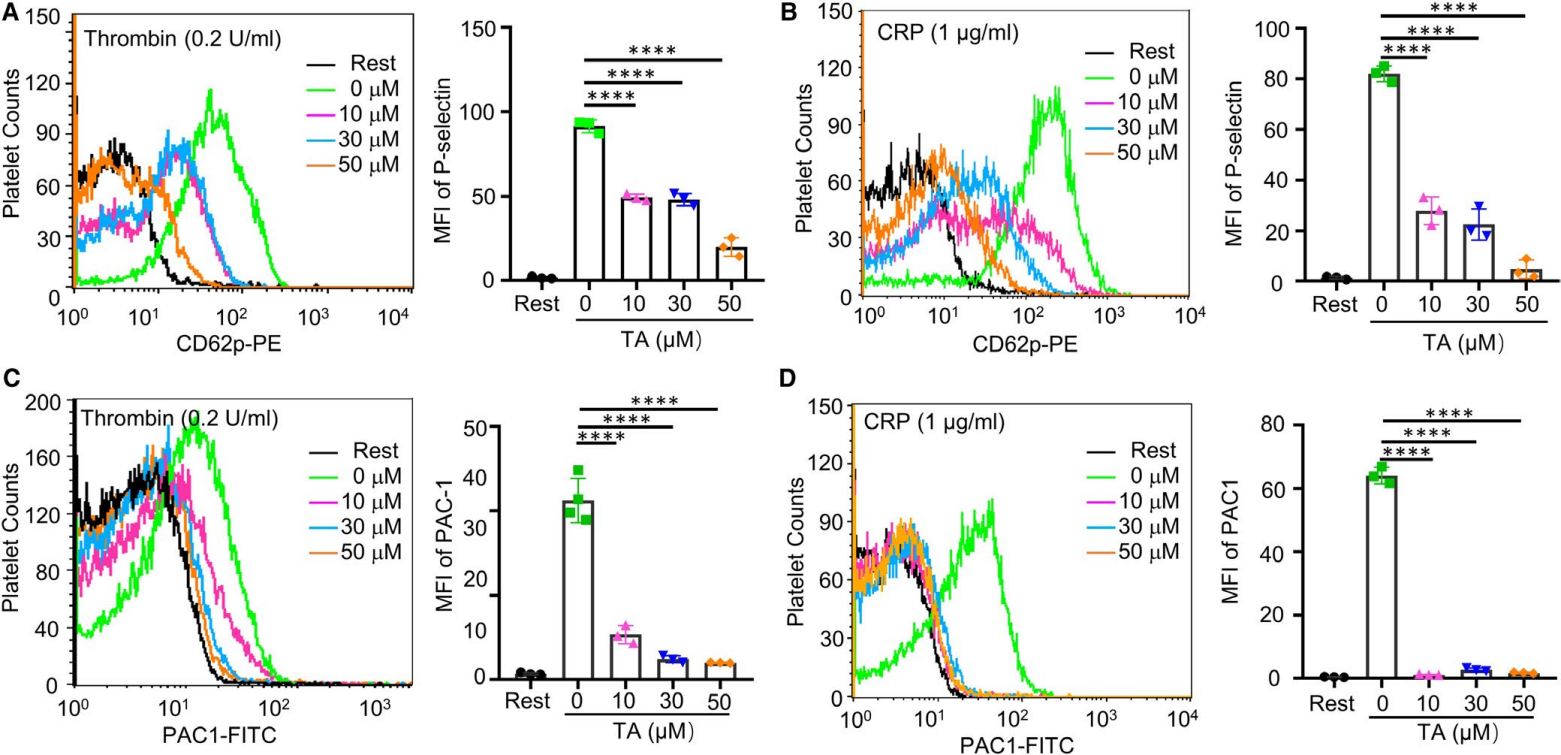
TA inhibits platelet P‐selectin expression and integrin αIIbβ3 activation. The P‐selectin expression (A, B) and integrin αIIbβ3 activation (C, D) were performed by flow cytometry. Human platelets were incubated with saline or TA (10, 30 and 50 μmol/L) for 10 min, and then stimulated with or without thrombin (0.2 U/mL) or CRP (1 μg/mL) in the presence of PE‐conjugated P‐selectin or FITC‐conjugated PAC‐1 antibody. The fluorescence intensity of PE‐CD62P or FITC‐PAC1 was recorded by flow cytometry. Overlay histograms and statistic diagrams were shown, n = 3 per group. Data were presented as means ± SD. Ordinary one‐way ANOVA, **P < .01, ****P < .0001

During activation, platelet granules are secreted, releasing pro‐thrombotic factors including P‐selectin. To evaluate the effect of TA on platelet activation, we used flow cytometry to detect platelet α granule content P‐selectin. Compared to the vehicle, TA treatment reduced thrombin‐ or CRP‐induced platelet P‐selectin expression (Figure 4A,B). To validate the inhibitory effect of TA on platelet activation, we further studied the effect of TA on integrin α_IIb_β_3,_ a pivotal activation receptor and major PDI substrate in platelets. Activated integrin α_IIb_β_3_, which indicates platelet inside‐out signalling, was labelled using FITC‐conjugated PAC‐1. Results showed that TA inhibited integrin α_IIb_β_3_ activation induced by thrombin (0.2 U/mL) and CRP (1 μg/mL) (Figure 4C,D). Therefore, TA inhibits both ITAM‐ or GPCR‐mediated platelet activation and attenuates platelet PDI signalling.

### 3.4 TA inhibits platelet spreading on immobilized fibrinogen and clot retraction

**FIGURE 5:**
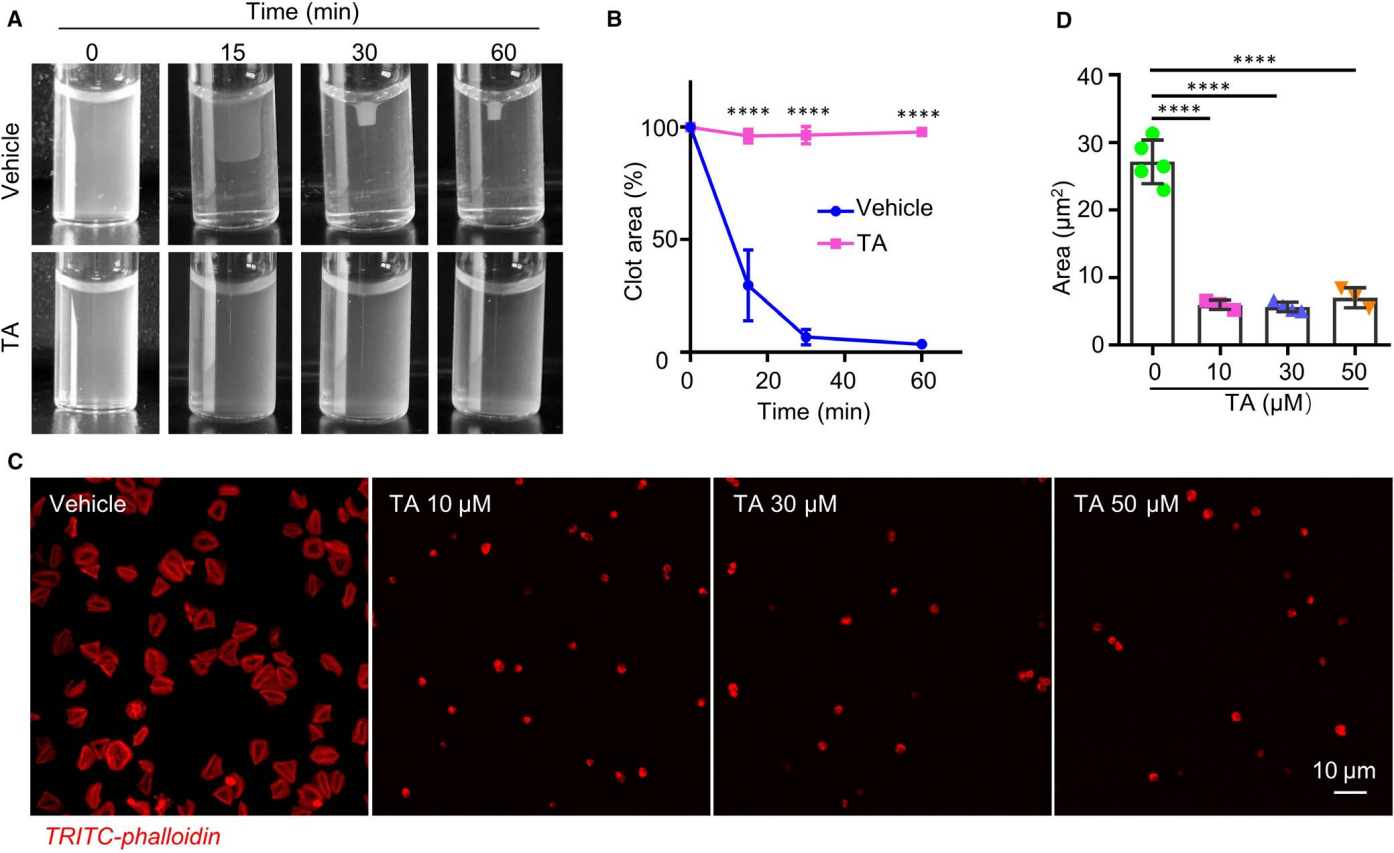
TA inhibits platelet clot retraction and spreading on immobilized fibrinogen. A, B, After pre‐incubation with vehicle or TA (30 μmol/L) for 10 min, gel‐filtered human platelets were stimulated with fibrinogen (2 mg/mL) and thrombin (1 U/mL), and the clot size was recorded at the indicated time point using a camera. Representative images at indicated time points were shown. B, The percentage of clot retraction area was quantified by the ratio of clot area to platelet suspension area. N = 3, per group. Data were presented as means ± SD. Two‐way ANOVA, ****P < .0001. C, TA (10, 30, 50 μmol/L)‐ or saline‐treated human platelets were placed on fibrinogen‐coated glass coverslips for 1 h at 37°C and stained with TRITC‐phalloidin. Representative images were shown. D, The statistical data were calculated by the mean surface area of individual platelet. N > 3 per group. Ordinary one‐way ANOVA, ****P < .0001

Our results showed that TA inhibits the activity of PDI and platelet inside‐out signalling. Activated integrin α_IIb_β_3_ mediates further outside‐in signalling that leads to irreversible platelet activation, which can be reflected by platelet spreading and clot retraction. To examine the effect of TA on platelet outside‐in signalling, isolated human platelets were pre‐treated with TA before thrombin stimulation. Continuous monitoring for 60 minutes showed that the area of the clot was significantly reduced in vehicle‐treated platelets. In contrast, the retraction of the clot was impaired in the TA‐treated (30 μmol/L) platelets (Figure 5A,B). Platelet spreading reflects the initial phase of outside‐in signalling following integrin activation. To test whether TA affects this event, platelets were incubated with TA and allowed to spread on immobilized fibrinogen, the canonical ligand of integrin α_IIb_β_3_. Immunofluorescence microscopy showed that TA (10, 30 and 50 μmol/L) treatment led to a significant reduction of platelet spreading area on immobilized fibrinogen compared with the vehicle (Figure 5C,D). Thus, TA inhibits the outside‐in signalling of platelet activation.

### 3.5 TA inhibits thrombus formation in vivo without increasing bleeding time

**FIGURE 6:**
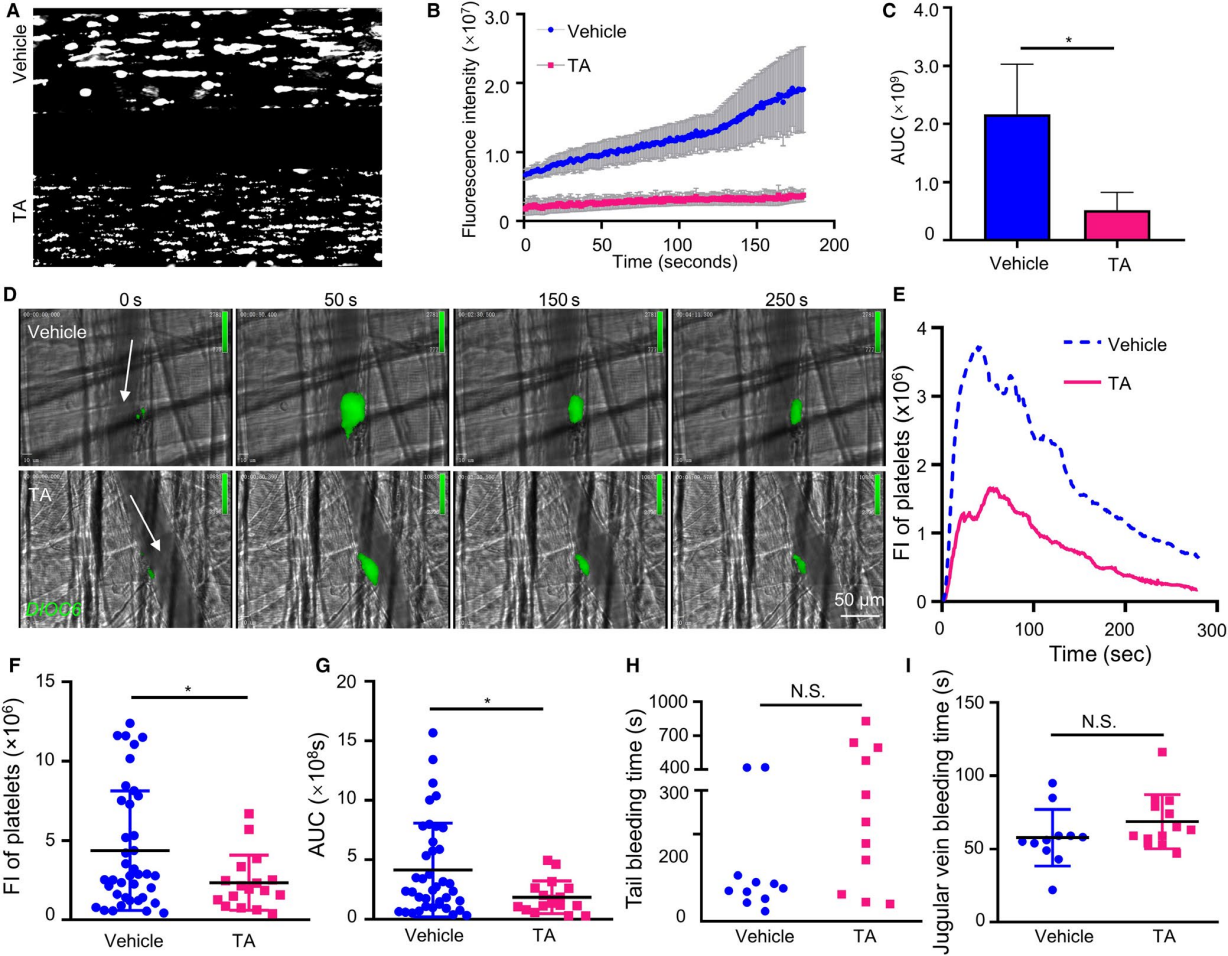
TA attenuates thrombus formation in vivo without prolonging bleeding time. A, TA inhibits the flow‐associated platelet adhesion on collagen. TA (50 µmol/L)‐ or vehicle‐treated human whole blood was labelled with calcein‐AM (10 µmol/L) for 30 min and perfused through the channels coated with collagen (100 µg/mL) at 10 dyne/cm^2^. B, Live fluorescence intensity was recorded. C, Area under curve (AUC) was calculated, vehicle versus TA, *P < .05, *t* test. D, Wild‐type C57B/L mice were administrated with TA (5 mg/kg) or saline by intraperitoneal injection before laser injury. The thrombus was visualized using 3,3′‐dihexyloxacarbocyanine iodide (DIOC6) staining and monitored in real‐time under an intravital microscope. Arrows indicate the directions of blood flow. E, DIOC6 fluorescence intensity curve showed dynamic changes in the size of the thrombi. F, Peak DIOC6 fluorescence intensity and G, thrombus sizes (area under the curve, AUC) were analysed. Number of thrombus: 14‐15 per group. Data were presented as means ± SD, unpaired *t* test, *P < .05. H, I, Effect of TA on bleeding time. C57B/L mice were administrated with TA (5 mg/kg) or saline by intraperitoneal injection. H, The tail was cut 3 mm, and the tail‐bleeding time was recorded. Data were presented as no line or error bar. N = 11 per group. I, The jugular vein bleeding time was also recorded after punctured with a needle. N > 11. Data were presented as means ± SD. NS, no statistical significance

Our results showed that TA inhibits platelet activation under static. However, whether the inhibitory effect of TA persists under flowing shear stress remains unclear. To evaluated whether TA inhibits platelet adhesion under flowing state, we used flow chamber to perfuse TA‐treated (50 μmol/L) human platelets through collagen‐coated channels. Live fluorescence was monitored real‐time. The result showed that TA significantly decreased the number of adherent platelets on collagen compared with vehicle (Figure 6A‐C).

To test whether the antiplatelet activity of TA can be translated to antithrombotic effects, we employed an in vivo mouse thrombosis model. Arterial thrombosis was induced in mouse cremaster arterioles by pulsatile argon laser and monitored on intravital microscopy. Platelet fluorescence‐labelled using DIOC6 was traced lively to quantify thrombus size dynamically. In mice receiving a single dose of TA (5 mg/kg ip) before thrombus induction, the area of thrombus was significantly reduced compared with the control group (Figure 6D,E). Further analyses showed that TA reduced the peak thrombus size by 46.3% and total thrombus area by 55.2% compared with the vehicle (Figure 6F,G, Video S1 and S2). These results suggested that TA inhibits the formation of arterial thrombosis in mice.

Mechanistically, PDI inhibitors target the high‐affinity transformation of integrin α_IIb_β_3_ and tend to retain initial platelet adhesion during vascular injury. They are therefore less likely to cause complete inhibition of integrin α_IIb_β_3_ and are therefore featured by lower bleeding risk. This notion is supported by the minimal interference of hemostasis by rutin, isoquercetin and ML‐359. To validate the safety of TA regarding the bleeding risk, we assessed the effect of TA on mouse bleeding time. First, a tail‐bleeding model was used. Administration of TA (5 mg/kg) 30 minutes before tail resection did not prolong the time to initial hemostasis compared with the vehicle (Figure 6H). Moreover, we used a venous bleeding model, in which mouse jugular vein was pierced to induce bleeding, to validate the result. The model allows direct observation of bleeding sites without causing massive adjacent tissue injury. Likewise, the TA‐treated group displayed comparable bleeding time as the control group (Figure 6I). In addition, the effect of TA administration into mice on platelet count or coagulation was also examined. The results showed that administration of TA (5 mg/kg) did not affect platelet count, APTT or PT (Figure S2A‐C). Together, TA inhibits thrombosis in vivo while preserving normal hemostasis.

## 4 DISCUSSION

In this study, we used an in silico computer‐based virtual screening and identified the plant polyphenol TA as a potential PDI inhibitor. We showed that TA binds PDI with high affinity and inhibits its activity and function in platelets. Moreover, TA inhibits thrombus formation without affecting hemostasis.

Previous studies indicated PDI as a novel antithrombotic target that may spare hemostasis. High‐throughput (HTP) screen has been used to identify PDI inhibitors from chemical banks. As natural polyphenols contribute a rich source of PDI inhibitors, we present an in silico approach to screen reported cardiovascular beneficial polyphenols. While the HTP method costs more resources and time, our method may benefit from decreased cost, enhanced flexibility and reduced time. Confined screening on natural polyphenols with long medical history may also reduce potential risk from novel synthetic molecules.^34^ In recent years, in silico screening based on molecular docking has been widely used in drug discovery. We used this technique as our first‐line screening tool to identify potential PDI inhibitors. Compared to reported PDI inhibitors including quercetin‐3‐rutinoside (binding constant *K*_D_ = 2.8 μmol/L),^9^ 12‐*O*‐tetradecanoylphorbol 13‐acetate (TPA) (*K*_D_ = 1.03 μmol/L),^35^ anti‐PDI mAb Clone 1D3 (*K*_D_ = 15 nmol/L),^36^ the *K*_D_ of TA with PDI was lower (below 1.68 nmol/L), suggesting a high affinity binding with PDI. Further elucidating the relationship between the characteristics of PDI inhibitors and their binding sites will facilitate understanding the underlying mechanism of action, thereby providing the theoretical basis to develop novel PDI inhibitors.

Multiple PDI family members, including PDI, ERp57, ERp72 and ERp5, have been shown to regulate platelet activation and thrombosis. By catalysing the reduction and isomerization of disulphide bonds in integrin α_IIb_β_3_, PDIs promote the conformational shift of the integrins, leading to full platelet activation. Because of different molecular structures, each PDI member recognizes different substrates and appears to play a distinctive role in platelet aggregation. For instance, the b′ subunit of ERp57 but not PDI interacts with calnexin and calreticulin. ERp57 and PDI also show different redox capacities to sulfhydryl oxidases. The b′ domain of PDI binds to substrates, the hydrophobic patches among the a°, a and a′ domains of ERp72 mediate substrate binding. Calnexin is a substrate to ERp57 but not ERp72. Endothelial PDI, ERp57 and ERp72 play a role in fibrin generation and contribute to thrombus formation.^1^, ^37^ Evidence from different studies indicates that the macro‐scaffold formed by multiple gallic acid groups in TA contributes to its biological activity. Compared to polyphenolic compounds containing fewer intragallogalloyl acyl groups, TA displays higher inhibitory effect on peptidyl‐prolyl cis/trans isomerase (Pin1), collagenase (Collagenase) and calcium‐activated chloride ions channel (Calcium‐activated chloride channel, CaCC).^38^, ^39^, ^40^ Also, TA inhibited the binding of PDI to the platelet surface integrin α_IIb_β_3_, suggesting that TA may interfere with the substrate‐binding centre of PDI. This phenomenon may be explained by the conformational change of the b′x domain secondary to the binding of TA to the active centre of PDI.^41^ Because PDI, ERp57, ERp72 and ERp5 play significant roles in regulating thrombosis, inhibiting multiple members may yield stronger antithrombotic effects.^1^ Regarding the potential synergy between different PDI members, inhibition of multiple PDIs may exert a stronger antithrombotic effect, but the potential bleeding risk warrants further validation. On the other hand, TMX1 of the PDI family exerts oxidase activity, mediates the inactivation of integrin α_IIb_β_3_ and inhibits platelet activation. It is not clear whether TA or existing PDI inhibitors may affect TMX1 function and raise the thrombotic risk.^42^ Exploring the effect of TA on different PDI members will shed more light on this topic.

Inhibiting PDI indirectly antagonizes integrin α_IIb_β_3_ full activation and non‐selectively inhibits platelet activation induced by agonists of different upstream signals.^43^ Unlike direct integrin α_IIb_β_3_ antagonists, PDI inhibitors spare hemostasis. Meanwhile, the minimal effect of PDI inhibitor on inside‐out signalling may also contribute to improved bleeding safety. In our study, we showed that TA inhibits PDI and multiple platelet pathways including GPCR and ITAM. Similarly, PDI knockout mice show attenuated thrombin and convulxin‐stimulated platelet aggregation.^44^ We noticed integrin α_IIb_β_3_, the major substrate of released PDI on the membrane surface during platelet activation, displays decreased activation after TA pre‐treatment.^45^ Recently, a TA‐coated material surface was reported to prevent thrombus formation by inhibiting fibrinogen conformational change under shear stress.^46^ Our flow cytometry experiments indicate that TA may directly inhibit the activation of integrin α_IIb_β_3_ activation under static flow conditions. Therefore, the negative effect of TA is attributed, at least partially but significantly, to the inhibition of PDI activity beyond its effect on fibrinogen conformation.

The target of PDI in platelet also includes GPIb that mediates platelet adhesion under a high shear rate. Recent studies have shown that PDI regulation of GPIb is involved in thrombotic inflammation and ischaemic organ damage.^47^ Inhibiting PDI may provide a novel approach to control thromboinflammation. Notably, TA has also been reported to protect acute ischaemic brain damage in rats^47^ and has protective effects against myocardial ischaemic injury.^48^ It may be inferred that PDI‐GPIb signalling is a potential target underlying the protective role of TA in ischaemic injury. Besides, PDI is also expressed in leucocytes and promotes cell activation. Hahm et al^4^ found that the stimulation of leucocytes with *N*‐Formylmethionyl‐leucyl‐phenylalanine (fMLF) peptide increased the amount of surface PDI, which mediates leucocyte migration by binding to and activating integrin α_M_β_2_. Endothelium‐derived PDI is involved not only in the regulation of thrombosis but also in the regulation of endothelial cell integrin α_V_β_3_ activation.^49^ The reported effect of TA in inhibiting endothelial inflammation and leucocyte activation may be mediated through leucocyte PDI.^50^, ^51^ Together, these results suggest that TA may exert pleiotropic protection against vascular inflammation by inhibiting PDI function in different cells involved. Also, inhibition of PDI may reduce the activation of coagulation factors.^10^ Therefore, multiple targets, including GPIb and integrin α_IIb_β_3_, may contribute to the antithrombotic effect of TA.

We showed that TA, at antithrombotic concentrations, did not induce platelet toxicity. Extracellular PDI tends to be a more favourable target in antithrombotic therapy because cytoplasmic PDI regulates protein synthesis and maintains normal cellular function, although whether this applies to platelets remains unknown.^52^ With an MW of 1701 g/mol, TA is unlikely to enter the cytoplasm when the cell membrane is intact, thereby minimizing the effect on cytoplasmic PDIs. Similarly, the PDI inhibitor rutin is considered as a safe antiplatelet agent due to its limited cell membrane permeability.^53^ In comparison, cell‐permeable PDI inhibitors, including PACMA‐31 and CCF642, may affect cytosolic PDI and cell viability.

Tannic acid did not dissociate platelets that had accumulated compared to direct antagonists of integrin α_IIb_β_3_. Thus, the risk of bleeding from inhibiting PDI tends to be lower than that of conventional antiplatelet drugs, even when both platelet and endothelial‐derived PDI were inhibited.^9^ Nevertheless, the results of different studies on the bleeding time of PDI knockout mice remain controversial, potentially due to differences in genetic background. The mouse tail‐bleeding experiment may also introduce artefacts from non‐specific destruction of perivascular tissues. To minimize the interference, we used a jugular vein puncture bleeding model. Both bleeding models yielded consistent safety of TA on mouse hemostasis. To fortify the safety profile of TA optimized for long‐term prevention and maintenance, effects of TA administration via different routes, of higher doses, and for a longer time are to be addressed in the future.

Interpretation of the coagulation parameters after TA treatment will uncover their safety basis for clinical application. Rated safe by FDA, TA is still used to treat diarrhoea, topical inflammation and dental caries.^54^ The expected blood concentration of TA according to clinical dosage is close to the antiplatelet levels. There was no obvious pathological change in liver, kidney and lung after oral administration of TA of 8750 mg/kg/d for one month in mice. Administration of 30 mg/kg/d TA in mice for six months led to no abnormalities in exercise, bodyweight, feeding and organ morphology.^55^ The price of TA is also much lower than that of PDI monoclonal antibodies as well as many existing PDI small molecule inhibitors. Thus, TA may provide a safe and cost‐effective choice to combat thrombosis. However, the bioavailability score of TA was lower than that of synthetic small molecule PDI inhibitors due to its larger molecular size. Abdominal, intravenous and even nasal administration of TA still achieved antioxidant, anti‐inflammatory and anti‐apoptotic effects although the absorption rate of TA is low.^56^, ^57^ Dissecting the distribution, metabolism of TA via different routes of administration will facilitate translating its protective role into clinical practice.

In summary, our results indicate that the plant polyphenol TA inhibits PDI activity, inhibits platelet activation and thrombosis. At concentrations and doses that exert antiplatelet antithrombotic effects, TA is non‐cytotoxic without affecting physiological hemostasis and is expected to develop a new safe antithrombotic drug.

## CONFLICT OF INTEREST

The authors confirm that there are no conflicts of interest.

## AUTHOR CONTRIBUTIONS

**Lijie Ren:** Conceptualization (lead); Data curation (lead); Formal analysis (equal); Funding acquisition (equal); Investigation (equal); Methodology (lead); Project administration (lead); Resources (supporting); Writing‐original draft (lead); Writing‐review & editing (equal). **Tao You:** Conceptualization (equal); Data curation (equal); Formal analysis (equal); Funding acquisition (supporting); Investigation (equal); Project administration (equal); Resources (supporting); Software (lead); Writing‐original draft (lead); Writing‐review & editing (equal). **Qing Li:** Conceptualization (equal); Data curation (equal); Formal analysis (equal); Methodology (supporting); Project administration (supporting); Software (equal); Writing‐original draft (supporting). **Guona Chen:** Methodology (supporting); Project administration (supporting). **Ziting Liu:** Project administration (supporting). **Xuefei Zhao:** Data curation (supporting); Methodology (supporting); Project administration (supporting). **Yinyan Wang:** Methodology (supporting); Project administration (supporting). **Lei Wang:** Methodology (supporting); Project administration (supporting). **Yi Wu:** Conceptualization (supporting); Methodology (supporting). **Chaojun Tang:** Conceptualization (supporting); Data curation (equal); Funding acquisition (equal); Project administration (supporting). **Li Zhu:** Conceptualization (lead); Data curation (lead); Formal analysis (equal); Funding acquisition (lead); Investigation (lead); Methodology (equal); Project administration (supporting); Resources (lead); Supervision (lead); Validation (lead); Visualization (lead); Writing‐original draft (supporting); Writing‐review & editing (lead).

## Supporting information

Fig S1

Fig S2

Video S1a

Video S2b

## Data Availability

The authors confirm that the data supporting the findings of this study are available within the article and its supplementary materials.
