# Supplementary figures and images for: Molecular docking‐assisted screening reveals tannic acid as a natural protein disulphide isomerase inhibitor with antiplatelet and antithrombotic activities
Source: J Cell Mol Med. 2020 Oct 30;24(24):14257–69. doi: 10.1111/jcmm.16043 (PMC7753999; doi:10.1111/jcmm.16043)

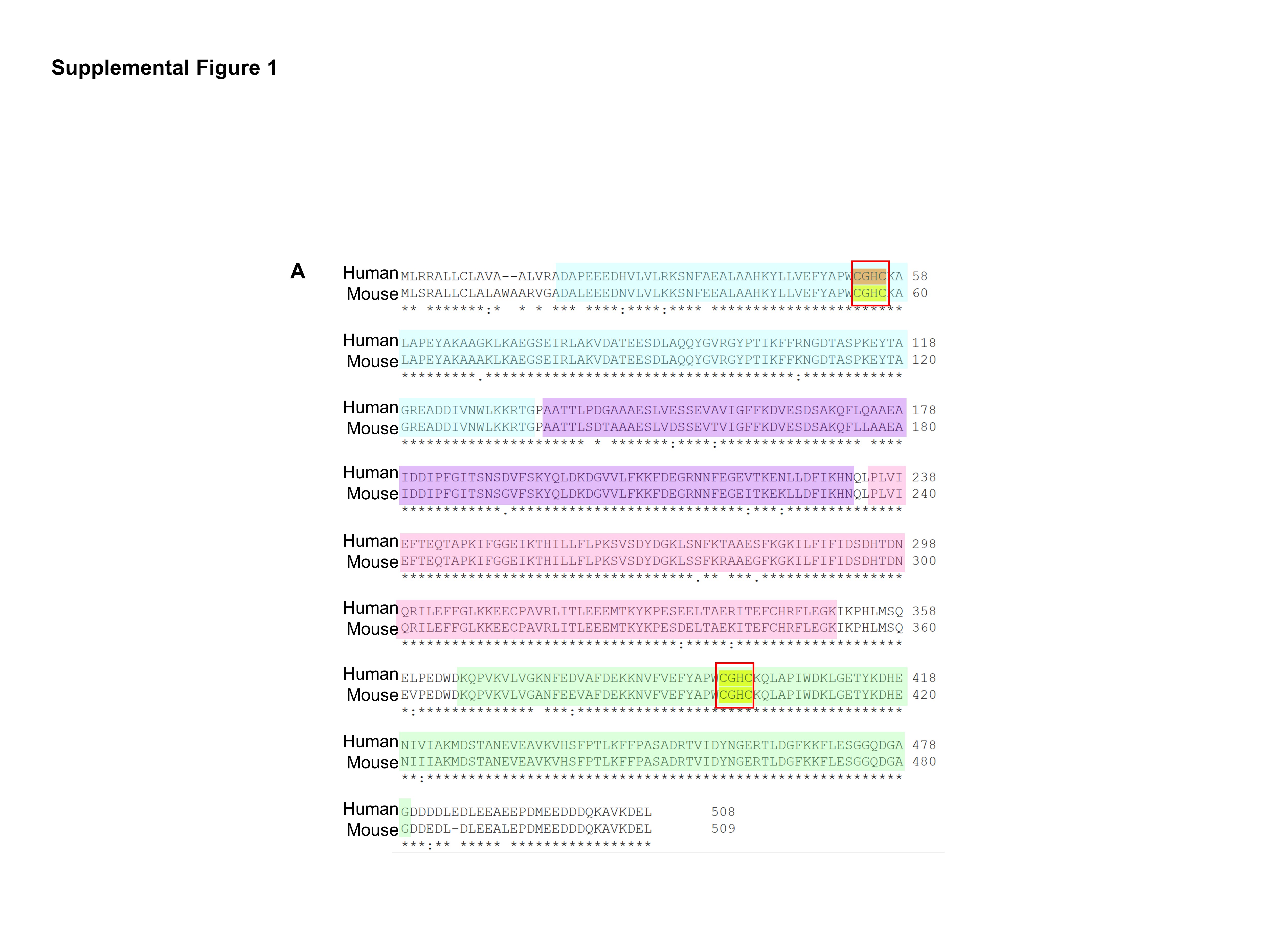

Supplement: Supplementary file 1 — Fig S1 [file JCMM-24-14257-s001.jpg]

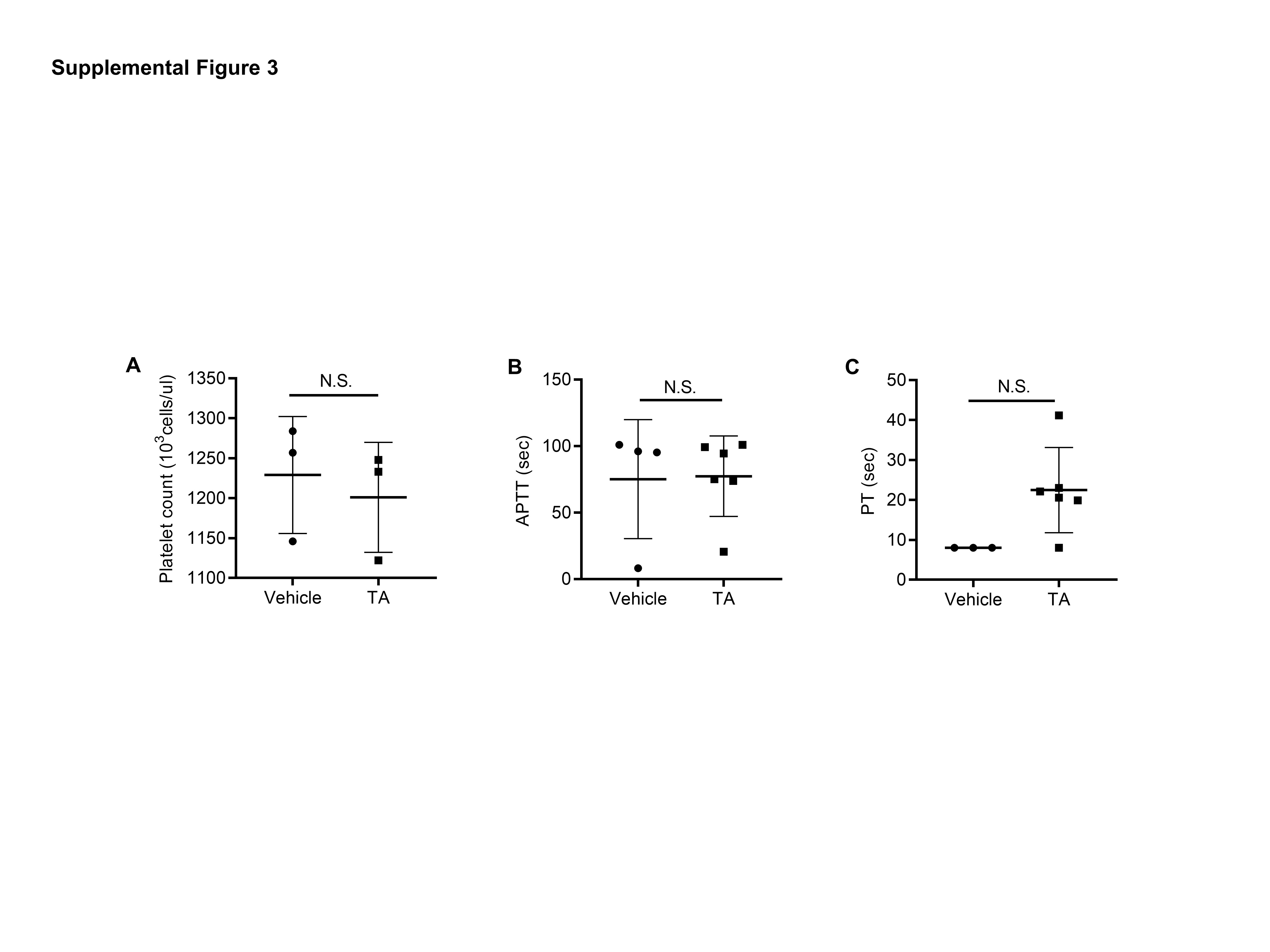

Supplement: Supplementary file 2 — Fig S2 [file JCMM-24-14257-s002.jpg]
